# Extract from Lectures on Experimental Pathology and Operative Physiology

**Published:** 1860-09

**Authors:** Claude Bernard


### EXTRACT FROM LECTURES ON EXPERIMENTAL PATHOLOGY AND OPERATIVE PHYSIOLOGY.

BY M. CLAUDE BERNARD.

Gentlemen:—In my first lecture I made you acquainted with the course which I proposed to carry out in the study of Experimental Pathology, and I endeavored to show you how difficult it was to enter on the study of so complex a science as that of Medicine, without having previously acquired a preliminary knowledge of other sciences less complicated in their nature; lastly, I endeavored to combat an opinion, too generally entertained, viz: that physiological phenomena belong to an order of facts entirely foreign to those which occur in the morbid state.

We shall now enter on the study of the symptoms peculiar to the pathological state, the agents which give rise to them. and those calculated to bring about their removal; lastly, we shall produce all the phenomena of disease by artificial means, and shall then endeavor to make them disappear.

But, before entering on this subject, it is indispensable to ascertain whether everything which occurs in a state of disease can be explained on physiological grounds; or whether disease has the peculiar property of originating, in the living being, laws altogether new, and of which we have not the slightest idea as existing in a state of health.

If, in the case of an adult in the full enjoyment of all his faculties, we ask ourselves what is the regulating agent, what the *primum mobile* of all physiological actions, we are constrained to reply that its seat is in the nervous system. It is to the nervous system that we owe both sensibility and voluntary motion, that two-fold source of all our relations with the external world; it presides over all organic functions, and it is my intention to prove to you, that while it is the origin of all the normal phenomena of life, it is also that of all pathological action.

In proportion as we ascend in the scale of animal life, we see the nervous system acquire greater development, and at the same time we observe that diseases become more frequent, more variable in their form, and more complicated in their nature. Why should this coincidence astonish us? Are not all our organs dependent directly on the nervous system? If we take, one by one, the different systems of the animal economy, it will be easy to show that all the symptom of the diseases to which they are liable, may be produced by direct irritation of their corresponding nerves. We can even give rise, in this way, to all the anatomical lesions by which they are characterized.

What, for instance, are the principal signs of the affections of the respiratory organs? Cough, dyspnœa, increased bronchial secretion; are not these the symptoms which most frequently proclaim their existence? Now all these phenomena can be produced at will by the direct excitation of the pneumogastric or certain other nerves; we can even call into existence the anatomical lesions incident to pleurisy and pericarditis. The causes of these morbid changes would therefore appear to be intimately connected with the nervous system. If we now turn our attention to the digestive apparatus, we shall soon be convinced that the physiologist possesses the same power relative to it, as he does in the case of the respiratory organs. By exciting the solar plexus and its efferent branches, we can determine both diarrhœa and dysentery, together with the anatomical lesions which habitually accompany them. Acute peritonitis has even been induced with all its consequences, as evidenced on opening the animal, by the presence of pus and false membrane in the peritoneal cavity.

Thus, then, a multitude of diseases may be brought into existence by a simple modification of the elements which the animal economy originally contains, without having recourse to the introduction of any new principle; and, if we were to examine the other systems of the body, results analogous in their nature would be obtained. Fever itself, that essentially medical symptom, can be excited by a mere mechanical irritation of the nervous system, and the products of inflammation, such as pus, false membranes, and plastic exudations, may, any or all of them, be called into existence in a similar way. In an animal, previously enfeebled, we can produce, directly, pleuritis with purulent deposit, by the simple division of the great sympathetic nerve; in order, however, to insure success in this experiment, it is absolutely necessary that the state or condition of the animal's health should be previously lowered.

It is, therefore, a fact, that the perverted state of the nervous system gives rise to a great variety of diseases, not only of a general, but also of a local character; deprive a muscle or a bone of its nervous supply, and you will have, as a consequence, fatty degeneration in the one case, and rickets in the other; in fact, if you tie the nerves, which enter the nutritive foramina of a bone, you will very soon see the cells of the lamellar structure increase in size, the vessels become more numerous, and all the phenomena of rickets follow in rapid succession; we can even bring about these results on part of a bone, without interfering with the remainder. This experiment has been successfully carried out, in the case of the lower jaw, by M. Schiff, of Berne.

But there exists in disease an immense number of other phenomena, which, at first sight, it appears impossible to produce by a simple lesion of the nervous system; I allude especially to the alteration or modification of the fluids of the body, which takes place in the course of certain maladies. Now, I am prepared to demonstrate that a vast number, if not all, of these morbid changes, are still to be traced to the action of the nervous system, and that they can be reproduced at pleasure by the physiologist. Among the various fluids of the body, the urine is that one the morbid changes of which have been the most carefully and completely investigated. Now you are perfectly aware, gentlemen, that albuminuria, polyuria, and diabetes are invariably produced by excitation of definite points of the medulla oblongata, the peculiar form of the perverted urinary secretion being determined by the particular portion which is acted on; it is in the case of diabetes especially that the importance of this experimental fact is fully brought out. It was supposed that in diabetic patients the morbid state created entirely new conditions in the economy, which gave rise to the pathological production called sugar; it is now, however, admitted on all hands that these phenomena are fully explained by the mere exaggeration of a normal function, in virtue of which glycose is generated in every individual even in a state of health. It is therefore evident that disease, in this case, is nothing more nor less than an exaggerated natural function.

There exists, however, a certain number of pathological products and morbid manifestations which we have not been able to imitate by the employment of artificial means. Shall we be able, at a later period, to connect these facts with those which already fall within the range of experimental physiology? Such is the scientific problem of the day. The question is, whether we shall, one day, be able to embrace pathology in its entire extent, within the compass of biological explanations, or whether we shall, in addition to all which we can imitate or explain, forever be compelled to recognize a *special principle*, mysterious in its nature, which we must acknowledge as a morbid or vital phenomenon?

Let us take, for example, eruptive fevers, small pox, scarlatina and measles; these are diseases, indeed, which it is impossible for us to produce without having recourse to a special virus. Shall we, one day, be able to realize this undertaking without the intervention of the peculiar animal poison on which they seem to depend? Must we not, first of all, solve the question, whether such diseases as those we have just specified can possibly exist in animals, even in those which approach most closely to the human species? Are they not in reality the exclusive property of the human organization?

Nothing is more difficult than to produce, through the agency of the nervous system, eruptive diseases in animals, the vitality of the skin of which is essentially different from that of man. We can, nevertheless, produce ecchymosis, congestions and glandular swellings; but it must always be borne in mind that each particular species of animal has its own peculiar diseases, which cannot be transmitted to a neighboring species, however closely allied they may be. Now man, in himself, presents a greater number of special diseases than all the other animals taken together.

Fortunately for physiology, such incompatibilities are rather the exception than the rule. Tubercle, cancer, and many other morbid productions, are found equally in animals and in man. Every disease which gives birth to morbid tissue is evidently a perversion of the nutritive function; now, who will venture to deny the influence which the nervous system exercises over this physiological act? We must, therefore, advance resolutely in the path which lies open before us, without allowing ourselves to be disheartened or intimidated by the difficulties of our science. But we must bear in mind that a disease is not characterized by one single symptom; it consists rather of a complete series of symptoms, standing to each other in the relation of cause and effect. It is, in fact a morbid evolution which offers a commencement, a middle, and an end; so that a skillful and practiced observer, on witnessing the first stage of a disease, can predict its probable termination. This is no doubt true; disease does not consist in an isolated symptom; it is a collection of symptoms. Now these reunions of morbid phenomena, we indubitably succeed in reproducing in animals. The functions of life are modified in various ways by a variety of different agents. Poisons determine real disease which present an unbroken chain of symptoms, consequent on the introduction into the system of the toxic agent. Here, therefore, we find an entire class of diseases which can be produced at will.

But setting aside this question of such vast dimensions, and to which we shall revert at a later period of our course, let us inquire whether, by mere surgical operations, by mere mechanical lesions, we can determine on the animals subjected to experiment, a certain number of morbid series. If you simultaneously remove the two kidneys of a dog, or simply tie the renal arteries, you immediately produce a general disturbance in the entire economy. The animal is powerless in expelling the execrementitial product which should pass off by this channel, and the whole system becomes gradually poisoned. At first the animal is not seriously affected; it continues to eat and digest its food for a certain lapse of time, which corresponds with the period of incubation in diseases; by and by it is attacked with vomiting and purging, shortly after which it dies.

What takes place in a case like this? Let us endeavor to explain it. During the first period the urea, which can no longer be eliminated by the kidneys, is expelled by the intestines. It is found, together with the salts of ammonia, in the animal's excrements, and even in the gastric juice. If this new mode of elimination could be prolonged indefinitely the animal would not become diseased—it would not die; but very soon the mucous membrane of the intestines, irritated by by the constant contact with the ammoniacal salts, gives rise to morbid changes. On the other hand, as long as the urea is eliminated by the intestines, it does not find its way into the blood. This fact has been demonstrated experimentally by MM. Prevost and Dumas, who have not, however, succeeded in explaining it. Now, at a later period, when the mucous lining of the intestine refuses to continue this function, which is altogether foreign to it, the urea finds its way into the blood, and the animal soon expires, comatose and convulsed.

When the cessation of the urinary secretion depends on the ligature of the renal arteries, this state of things may sometimes be obviated by removing the ligatures; the self-same thing would also take place in man, if there existed an obstacle to the passage of the urine, and if it were possible to remove that obstacle; but in all cases in which the kidneys have been removed, death has always supervened. The destruction of the animal has been the invariable termination of the morbid series.

Here, then, we have a disease which can be artifically produced; but there are many others, the causes of which are agents existing exterior to the body; contagious affections belong to this class. The fact has been experimentally proved in the case of the peri-pneumonia of horned cattle, by the establishment of a communication between two cow-houses, the one containing healthy, the other diseased cattle. In the inclosure, which, in the first instance, contained only healthy cattle, several cases of this peculiar affection occurred in succession.

But independently of these various causes, the action of which I am far from being disposed to deny, internal accidents occur, and give origin to various affections in the economy. This has been experimentally proved; if you remove both the kidneys of an animal, it dies; if you remove, however, only one kidney, the animal continues to live; the organ becomes enlarged, and plays both its own part and that of its absent fellow; a fact which can be easily ascertained by opening the animal a certain time after the operation has been performed. But if, instead of removing the kidneys, you simply make a division of their nerves, the animal dies. During the first few days which follow the operation, albuminuria is produced; shortly after, inflammation of the kidneys sets up; they then mortify and become decomposed; so that, finally, they act on the economy like a septic poison, which inevitably leads to death. Such I consider to be the natural explanation of this *apparently* mysterious fact.

I now presume that I have established the initial proposition: Not only can we succeed in producing morbid symptoms in animals by artificial means, but even actual diseases, with their complete chain of results. Pathology, regarded from this point of view, combines the rèsources of physiology with those derived from clinical observation.

